# Efficient Removal of Tetracycline from Aqueous Media with a Fe_3_O_4_ Nanoparticles@graphene Oxide Nanosheets Assembly

**DOI:** 10.3390/ijerph14121495

**Published:** 2017-12-01

**Authors:** Xinjiang Hu, Yunlin Zhao, Hui Wang, Xiaofei Tan, Yuanxiu Yang, Yunguo Liu

**Affiliations:** 1 College of Environmental Science and Engineering, Central South University of Forestry and Technology, Changsha 410004, China; 2 Faculty of Life Science and Technology, Central South University of Forestry and Technology, Changsha 410004, China; 3 College of Natural Resources and Environment, South China Agricultural University, Guangzhou 510642, China; 4 Institute of Bast Fiber Crops, Chinese Academy of Agricultural Sciences, Changsha 410205, China; wanghuii623@163.com (H.W.); gym204@126.com (Y.Y.); 5 College of Environmental Science and Engineering, Hunan University, Changsha 410082, China; tanxf@hnu.edu.cn (X.T.); hnuese@126.com (Y.L.); 6 Key Laboratory of Environmental Biology and Pollution Control (Hunan University), Ministry of Education, Changsha 410082, China

**Keywords:** tetracycline, antibiotic wastewater, Fe_3_O_4_@GO, humic acid, ionic strength

## Abstract

A readily separated composite was prepared via direct assembly of Fe_3_O_4_ magnetic nanoparticles onto the surface of graphene oxide (GO) (labeled as Fe_3_O_4_@GO) and used as an adsorbent for the removal of tetracycline (TC) from wastewater. The effects of external environmental conditions, such as pH, ionic strength, humic acid (HA), TC concentration, and temperature, on the adsorption process were studied. The adsorption data were analyzed by kinetics and isothermal models. The results show that the Fe_3_O_4_@GO composite has excellent sorptive properties and can efficiently remove TC. At low pH, the adsorption capacity of Fe_3_O_4_@GO toward TC decreases slowly with increasing pH value, while the adsorption capacity decreases rapidly at higher pH values. The ionic strength has insignificant effect on TC adsorption. The presence of HA affects the affinity of Fe_3_O_4_@GO to TC. The pseudo-second-order kinetics model and Langmuir model fit the adsorption data well. When the initial concentration of TC is 100 mg/L, a slow adsorption process dominates. Film diffusion is the rate limiting step of the adsorption. Importantly, Fe_3_O_4_@GO has good regeneration performance. The above results are of great significance to promote the application of Fe_3_O_4_@GO in the treatment of antibiotic wastewater.

## 1. Introduction

In recent years, pharmaceuticals and personal care products (PPCPs) have been frequently detected in the environment, and they have attracted scrutiny as a new type of pollutant that threatens the biosphere and human health [1,2]. Antibiotics are one of the main PPCPs. Since penicillin was discovered in 1929, antibiotics have been used extensively to improve the health of animals and humans, and antibiotics have also been used as growth promoters for livestock, bees, aquatic products and other aquaculture industries [3,4]. Globally, the average total annual use of antibiotics is approximately 200,000 tons [4,5]. At present, the most widely used antibiotics are the sulfonamides (SAs) [6], quinolones (QNs) [7], macrolides (MALs) [7], and tetracyclines (TCs) [8], among which TCs are some of the most frequently detected antibiotics in water [9]. In the process of biological metabolism, the vast majority of TCs is discharged through feces and urine directly from the body and, ultimately, into the water environment [10]. This damages the aquatic ecosystem and induces the generation of resistant genes in the environment, resulting in continuous pollution [11]. Thus, there is an urgent need to develop a low-cost, high-efficiency and productive removal technology.

TC removal technologies mainly include biological, adsorption, photolysis and advanced oxidation methods [11]. Because of its high efficiency, lack of byproducts and other characteristics, adsorption methods have been widely used in the removal of TCs from water [8,12,13,14]. Adsorbent materials currently being studied include graphene oxide (GO) [15], magnetite [9,12,13], goethite [16], birnessite [17], and chitosan [18]. GO is a two-dimensional material consisting of a single layer of carbon atoms arranged in six-membered rings and is one of the thinnest known 2-D materials [19]. GO has unique physical and chemical properties and has become a major research focus. The oxygen-containing groups on the surface of GO can be used as an adsorbent for the adsorption, extraction and separation of antibiotics, thus rendering GO of great significance for environmental protection [20].

Because of the small particle size and hydrophilic groups on the surface, GO-based adsorbent materials can be easily dispersed in water [19]. After completing the adsorption process, the material needs to be separated from the aqueous solution by filtration or centrifugation [21]. These separation methods have a high cost, require complex operation steps, and are time consuming, among other shortcomings. Magnetic separation technology has gradually attracted the attention of several scientists and technical researchers [8,22]. It has been used in the fields of medicine, cell biology, analytical chemistry, mining and environmental technology [23]. Some magnetic materials, such as Fe_3_O_4_ and γ-Fe_2_O_3_, can be coupled with GO to impart magnetic properties to the adsorbent, and the adsorbent can then be separated from the reaction medium through the use of a magnetic field. A composite material prepared by coupling Fe_3_O_4_ magnetic nanoparticles with GO combines the excellent adsorption characteristics of GO and the easy separation of magnetic materials.

In this study, Fe_3_O_4_ magnetic nanoparticles were coupled with GO to prepare magnetically separable Fe_3_O_4_@GO composites, and their physical and chemical properties were characterized. The adsorption properties of the coupling material toward TCs in water were studied. The effects of pH, ionic strength, humic acid (HA), TC concentration, and temperature on the adsorption process were studied. An adsorption mechanism was developed based on kinetics and isothermal models, and the reusability of the coupling material was studied.

## 2. Materials and Methods

### 2.1. Preparation of Fe_3_O_4_@GO

#### 2.1.1. Preparation of Preoxidized Graphite

Graphite powder (particle size ≤ 30 μm) was purchased from Tianjin Hengxin Chemical Preparation Co., Ltd. (Tianjin, China). Graphite powder (30 g), K_2_S_2_O_8_ (25 g) and P_2_O_5_ (25 g) were added to concentrated sulfuric acid (98%, 120 mL) and allowed to react at 80 °C for 4.5 h. After cooling this mixture to room temperature, ultrapure water (1000 mL) was added, and the resultant system was allowed to stand for 12 h. The product was washed to neutral pH to obtain preoxidized graphite [24].

#### 2.1.2. Preparation of GO

The obtained preoxidized graphite (6 g) was added to concentrated sulfuric acid (98%, 240 mL) and NaNO_3_ (5 g) and KMnO_4_ (30 g) were then added. The solution was allowed to react in a 0 °C ice bath for 4 h and heated to 35 °C for 2 h, and then ultrapure water (500 mL) was added. The solution was heated to 98 °C for 1 h, and then ultrapure water (1000 mL) and concentrated H_2_O_2_ (40 mL) were added at room temperature. The reaction was continued for 2 h. The resulting product was washed with a large amount of water to neutral pH, and water was then added to obtain a 5 mg/mL suspension. Finally, ultrasonic dispersion was carried out at 50 °C to obtain an aqueous suspension of GO [24,25].

#### 2.1.3. Preparation of Fe_3_O_4_@GO

FeCl_3_·6H_2_O (0.05 mol) and FeSO_4_·7H_2_O (0.025 mol) were dissolved in ultrapure water (200 mL) at room temperature. The obtained mixed solution of ferric ions and ferrous ions was added to the above GO suspension (500 mL), and the mixture was rapidly stirred for 2 min in an 85 °C water bath. Then, a NaOH solution (100 g/L) was quickly added to the solution to adjust the pH to approximately 10. The solution was stirred for 45 min, and the resulting mixture was cooled. The precipitate was separated and washed to neutral pH with ethanol and Milli-Q water; water was then added to the product to obtain a 3.50 mg/mL Fe_3_O_4_@GO suspension.

### 2.2. Characterization

The morphology of the samples (the Fe_3_O_4_@GO suspension was dropped on a copper network and dried at room temperature) was examined by high-resolution transmission electron microscopy (HRTEM, Tecnai-G2-F20, FEI, Hillsboro, OR, USA) with an acceleration voltage of 200 kV. The samples were analyzed by Fourier transform infrared spectroscopy (FTIR, Magna-IR 170, Thermo Fisher Scientific, Waltham, MA, USA). The samples were prepared using the potassium bromide (KBr) powder compression method. The XRD pattern of the Fe_3_O_4_@GO was measured on a diffractometer with Cu Ka radiation source (40 kV, 250 mA) and equipped with a Kβ filter (D/max-2500, Rigaku, Tokyo, Japan). The Fe_3_O_4_@GO suspension was dried at 60 °C in oven and ground into a powder, then the magnetic properties of the sample were measured by a MPMS-XL-7 vibrating sample magnetometer (Quantum Design Instruments, San Diego, CA, USA), and the hysteresis loop of the dried samples was obtained. TG-DSC curves were performed using a thermal analyzer (SDT Q600, TA, New Castle, DE, USA) under a nitrogen atmosphere from room temperature to 1200 °C in an alumina crucible and the (sample mass 7.77 mg, flux rate 100 mL/min, heating rate 10 °C/min). This thermal analyzer can automatically correct the buoyancy effects. The surface elements were analyzed using X-ray photoelectron spectroscopy (XPS, ESCALAB 250Xi, Thermo Fisher Scientific, Waltham, MA, USA) with an Al Kα source (resolution of 0.5 eV).

### 2.3. Adsorption Experiment

The TC stock solution (1 g/L) was prepared by dissolving TC (1000 g) in a 100 mL dilute hydrochloric acid solution (0.1 M), transferring the solution to a 1000 mL volumetric flask and adding ultrapure water to a predefined volume. The different concentrations of TC solution used in the experiment were all diluted using this stock solution. All solutions were stored in a dark and cool place. The pH of the solution was adjusted with a 0.1–0.5 M NaOH or HNO_3_ solution. The solution was treated with ultrapure water (18.25 MΩ/cm) in the experiment. All adsorption experiments were carried out by adding 1 mL of a 3.50 ± 0.16 mg/mL Fe_3_O_4_@GO suspension to a conical flask containing 50 mL of a TC solution and placing the conical flask in a rotary shaker for 24 h at 150 rpm. After the reaction, Fe_3_O_4_@GO was separated from the solution using a magnet. The concentration of TC in the supernatant was determined by ultraviolet spectrophotometry (UV754N). The amount of adsorption was calculated by using the following formula:

(1) $$q_{e}=\frac{\left(C_{0}-C_{e}\right)V}{W}$$

where *q*_e_ (mg/g) represents the amount of adsorption at equilibrium; *C*_0_ and *C*_e_ (mg/L) represent the initial concentration and the equilibrium concentration of TC in the solution, respectively; *V* (L) represents the volume of the solution; and *W* (g) represents the dosage of the adsorbent.

### 2.4. Desorption Experiment

To further evaluate the regeneration and reusability of Fe_3_O_4_@GO, a desorption experiment was carried out. First, 3.50 mg/L Fe_3_O_4_@GO (3 mL) was added to a 10 mg/L pH 4.0 TC solution (150 mL). The reaction was oscillated at 150 rpm in a 30 °C water bath for 24 h. The remaining concentration of TC in the supernatant was measured, and the adsorption capacity was calculated. The Fe_3_O_4_@GO/TC complex was isolated from the reaction solution with a magnet, and the solid was washed several times with a NaOH solution and ultrapure water. The collected adsorbent was reintroduced into 150 mL of a 10 mg/L pH 4.0 TC solution, and the regeneration performance of Fe_3_O_4_@GO was investigated under the same conditions. The same experiment was carried out four times under the same conditions.

## 3. Results and Discussion

### 3.1. Characterization

**Figure 1:**
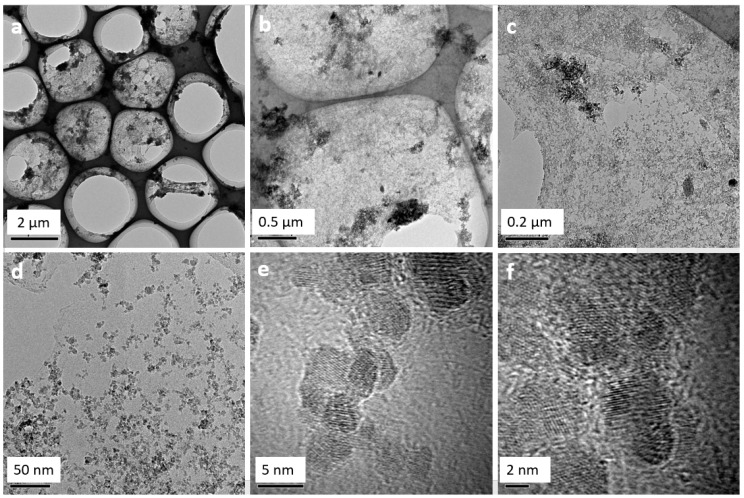
HRTEM image of Fe_3_O_4_@GO at different magnification; (**a**) 2100×, (**b**) 7000×, (**c**) 19,500×, (**d**) 71,000×, (**e**) 790,000×, (**f**) 1,050,000×.

Figure 1 shows the morphology of Fe_3_O_4_@GO at different resolutions, as obtained with HRTEM. It can be seen that several Fe_3_O_4_ magnetic nanoparticles with a particle size of ~5 nm are attached to the surface of GO. In addition, there are multiple folds on the surface of the Fe_3_O_4_@GO material. 

**Figure 2:**
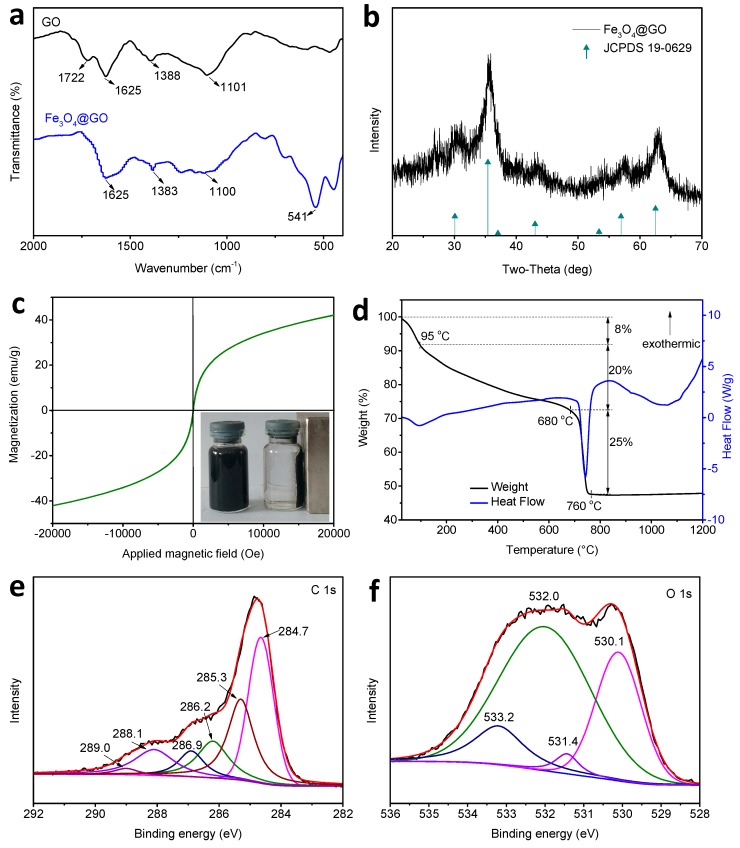
Characterization: (**a**) FTIR spectra of GO and Fe_3_O_4_@GO; (**b**) XRD pattern of Fe_3_O_4_@GO; (**c**) Magnetization curve (the right inset shows the magnetic separation of Fe_3_O_4_@GO); (**d**) TG-DSC curves; (**e**) C1s and (**f**) O1s XPS spectra of the Fe_3_O_4_@GO.

Figure 2a presents the FTIR characterization of GO and Fe_3_O_4_@GO. The characteristic peaks of GO appear at 1722 cm^−1^ (C=O in carboxyl group), 1625 cm^−1^ (C=C in the aromatic ring) [26], 1388 cm^−1^ (C–OH functional group) [27], and 1101 cm^−1^ (vibration of an epoxide group) [28]. For the Fe_3_O_4_@GO, the absorption peak for C=O in the carboxyl group has disappeared and the peaks of C–OH and epoxide group are shifted to 1383 cm^−1^ and 1100 cm^−1^, respectively, which may be due to the fact that Fe_3_O_4_ encapsulates the groups on the surface of GO [29]. The peak at 541 cm^−1^ corresponds to the stretching vibration of the Fe–O bond [29]. Figure 2b illustrates the XRD spectrum of Fe_3_O_4_@GO. Seven diffraction peaks at 2θ = 30.10°, 35.42°, 37.05°, 43.05°, 53.39°, 56.94°, and 62.52° are detected, which matches well with the indexed peaks of Fe_3_O_4_ (JCPDS 19-0629) [30,31]. Figure 2c shows the magnetization of Fe_3_O_4_@GO in the range of ±20 kOe. It can be seen that the hysteresis loops of the Fe_3_O_4_@GO sample are essentially overlapped in both the S-shape and when passing through the origin. The saturated magnetization (Ms) of the sample is 42.14 emu/g, which is lower than that of the pure Fe_3_O_4_ (74.0 emu/g) reported by Huang et al. [2]. This may be due to that the Fe_3_O_4_@GO is composed of the non-magnetic GO and the magnetic Fe_3_O_4_. The insert on the right of Figure 2b shows that with an external magnetic field, the Fe_3_O_4_@GO adsorption-treated sample was adsorbed on the “magnetized” wall of the reaction vessel in a short period. Thus, Fe_3_O_4_@GO exhibits superparamagnetism and meets the requirements of magnetic separation. The thermal stability of Fe_3_O_4_@GO was determined by TG-DSC curves. 

Figure 2d presents the TG-DSC curves of the Fe_3_O_4_@GO composite, revealing the residual mass as a function of temperature. From the start of heating to 95 °C, the 8% weight loss is the evaporation of physically adsorbed water in the sample. From 95 °C to 680 °C, Fe_3_O_4_@GO has a mass loss of 20 wt% because of the functional group loss from the graphene layer. From 680 °C to 760 °C, there is a significant mass loss stage (25%) because of the thermal decomposition of the carbon skeleton. The elemental composition and chemical valence states of Fe_3_O_4_@GO were further analyzed by XPS. Figure 2e shows the C 1s XPS pattern for Fe_3_O_4_@GO. The carbon atoms are highly oxidized and are present in five distinct chemical environments, including C=C (284.7 eV), C–C (285.3 eV), C–O (286.2 eV), C–O–C (286.9 eV), C=O (288.1 eV), and O–C=O (289 eV) [32,33]. Figure 2f is the O 1s XPS pattern for Fe_3_O_4_@GO. The peak at 530.1 eV corresponds to O–Fe in the magnetic nanoparticles, while the peaks at 531.4, 532, and 533.2 eV correspond to O–H, O–C, and O=C groups, respectively [34].

### 3.2. Effect of pH

**Figure 3:**
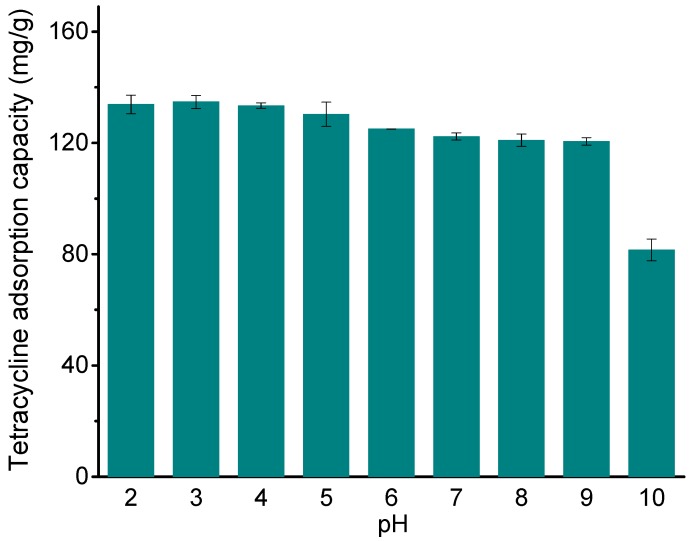
Effect of the initial pH on TC removal using Fe_3_O_4_@GO: *m*/*V* = 70 g/L, *C*_0(TC)_ = 10 mg/L, *T* = 303 K, *t* = 24 h.

The TC adsorption properties of Fe_3_O_4_@GO were investigated at initial pH values between 2 and 10 in the reaction solution. The results are shown in Figure 3. When the pH value is between 2 and 7, the adsorption capacity of Fe_3_O_4_@GO toward TC decreases gradually as the pH value increases. When the pH value is greater than 9, the adsorption capacity decreases rapidly. The results show that an acidic environment is more favorable for the enrichment of TC on Fe_3_O_4_@GO. Similar results were also found for oxytetracycline adsorption onto magnetite [12]. This is mainly because the pH value of the solution affects the surface charge of the adsorbent and the form of TC in the solution [35]. At low pH, the surface of Fe_3_O_4_@GO is positively charged due to the protonation reaction [36]. With increasing pH values, the surface of Fe_3_O_4_@GO becomes negatively charged due to the deprotonation reaction [37]. In addition, the pH value affects the ionization degree of the TC molecules. TC has three chemically distinct acidic functional groups: carboxymethyl (pK_a_ = 3.30), phenolic diketone (pK_a_ = 7.68) and dimethylamine cation (pK_a_ = 9.69) [38,39]. Thus, when the pH of the solution is less than 3.30, H_4_TC^+^ ions are the dominant form of TC in the solution. When the pH is in the range of 3.30–7.68, TC can be thought of as a mixture of a dimethylamino group and a negatively charged hydroxyl group, with H_3_TC as the dominant form. When the pH is in the range of 7.68–9.69, H_2_TC^−^ ions are the dominant form of TC. When the pH value of the solution is greater than 9.69, TC is mainly in the form of HTC^2−^. At low pH, both Fe_3_O_4_@GO and TC are positively charged, while the highest sorption is observed, which may be due to that the covalent type/inner sphere type interactions are stronger than the non-specific electrostatic interactions of Fe_3_O_4_@GO and TC [12,14]. 

Rakshit et al. [13] and Figueroa et al. [40] also reported similar results, and this speculation was evidenced using an in situ attenuated total reflectance-Fourier-transform infrared (ATR-FTIR) spectroscopy. As the pH increased, H_4_TC^+^, H_3_TC, H_2_TC^−^ and other forms of TC in the solution are mainly captured by Fe_3_O_4_@GO through electrostatic, hydrogen, π–π bonds, and ligand exchange reaction [12]. When the pH is higher than 9, the electrostatic repulsion between the negatively charged HTC^2−^ and the negatively charged Fe_3_O_4_@GO increases, resulting in the rapid decrease in TC adsorption.

### 3.3. Effect of Ionic Strength

**Figure 4:**
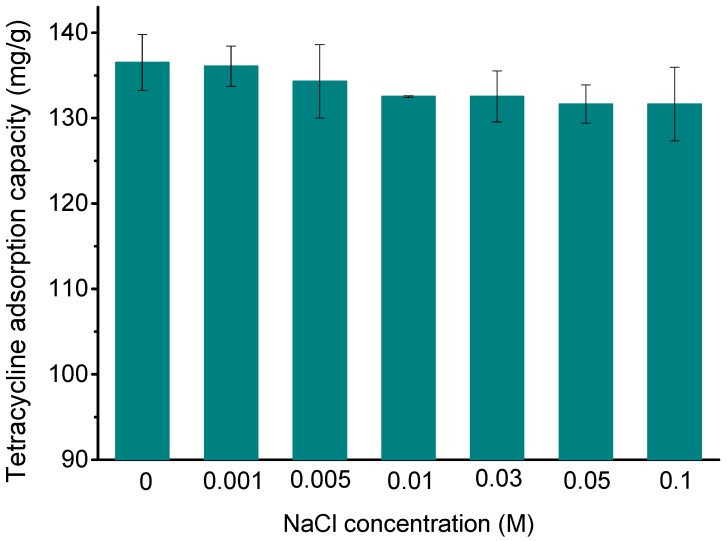
Effects of ionic strength on TC adsorption by Fe_3_O_4_@GO: pH = 4.0, *m*/*V* = 70 g/L, *C*_0(TC)_ = 10 mg/L, *T* = 303 K, *t* = 24 h.

In addition to contaminants, industrial wastewater also contains a variety of salts, and the presence of these salts may affect the removal of pollutants [3]. In this study, the effects of ionic strength on the removal of TC by Fe_3_O_4_@GO were investigated by using a series of TC solutions containing different concentrations of NaCl (0–0.1 M). It can be seen from Figure 4 that the differences of the adsorption capacity under different ionic strength are not significant. Rakshit et al. [12] also reported the same effect of ionic strength on TC adsorption onto magnetite. Generally, no effect or increasing sorption with increase of ionic strength indicates inner sphere type surface complexation [12,41]. Therefore, this result further supports the fact that the adsorption process of TC onto Fe_3_O_4_@GO is inner sphere type. Although the Na^+^ and Cl^−^ in the solution may interact with the TC molecular and the Fe_3_O_4_@GO, the adsorption efficiency is almost independent of the ionic strength, indicating that the surface complexation of TC with Fe_3_O_4_@GO is very strong.

### 3.4. Effect of HA Concentration

**Figure 5:**
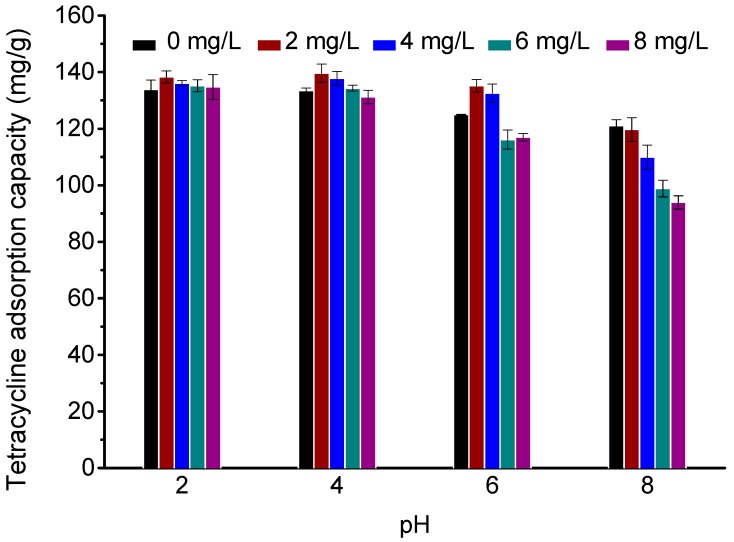
Effects of different humic acid concentration (2, 4, 6, and 8 mg/L) on TC adsorption onto Fe_3_O_4_@GO under various pH (pH = 2, 4, 6, and 8), *m*/*V* = 70 g/L, *C*_0(TC)_ = 10 mg/L, *T* = 303 K, *t* = 24 h.

HA is common in aqueous solutions and often coexists with antibiotics in wastewater. HA molecules contain carboxyl, phenolic hydroxyl, methoxyl, amido and other functional groups, which can interfere with the interactions between TC and an adsorbent. Therefore, it is of great significance to study the effect of HA on the adsorption process of TC. The effect of different concentrations of HA (0, 2, 4, 6, and 8 mg/L) on the adsorption of TC on Fe_3_O_4_@GO under different pH conditions (HA was firstly added into the TC solutions and then the pH values were measured and adjusted to 2, 4, 6, and 8, respectively) is shown in Figure 5. At pH values of 2, 4, and 6, the presence of 2 mg/L HA greatly promotes the adsorption of TC, while the adsorption capacity decreases with increasing HA concentration. This mainly occurs because at low concentrations, HA is adsorbed on the surface of Fe_3_O_4_@GO by hydrogen bonding, electrostatic attraction and π–π conjugation, and the groups on the HA molecules can integrate with the TC molecules. This is the same as increasing the number of adsorption sites on the Fe_3_O_4_@GO surface. When the added HA concentration exceeds 2 mg/L, the amount of free-moving HA molecules in the solution increases, which competes with the adsorbent to adsorb TC ions in the solution, leading to a decrease of the Fe_3_O_4_@GO adsorption capacity [31]. At pH 8, the addition of HA decreases TC adsorption. This is mainly due to the adsorbed HA being deprotonated and negatively charged under alkaline conditions; as H_2_TC^−^ is the dominant form of TC at this pH value, there is a large electrostatic repulsion between the two, which disrupts TC adsorption.

### 3.5. Adsorption Kinetics

Adsorption kinetics is an important parameter describing the adsorption rate of an adsorbate at the solid-liquid interface. Therefore, kinetics experimental data were simulated using pseudo-first-order, pseudo-second-order, two-compartment, intraparticle diffusion, and Boyd models in this study [42,43,44,45,46,47]. The nonlinear equation of the pseudo-first-order model is presented as Equation (2):

(2) $$q_{t}=q_{e,1}\left(1-e^{k_{1}t}\right)$$

The nonlinear equation of the pseudo-second-order model is given by Equation (3):

(3) $$q_{t}=\frac{q_{e,2}^{2}k_{2}t}{1+q_{e,2}k_{2}t}$$

The two-compartment model can be expressed as Equation (4).

(4) $$\frac{q_{t}}{q_{t=\infty }}=F_{\mathrm{fast}}\left(1-e^{-\frac{t}{k_{\mathrm{fast}}}}\right)+F_{\mathrm{slow}}\left(1-e^{-\frac{t}{k_{slow}}}\right)$$

The intraparticle diffusion model is represented as Equation (5):

(5) $$q_{t}=k_{p}t^{0.5}+C$$

where, *q*_t_ and *q*_t = ∞_ (mg/g) are the adsorption capacities at time *t* and adsorption equilibrium, respectively; *k*_1_ and *k*_2_ (g/mg min) are the pseudo-first-order and pseudo-second-order kinetics adsorption rates, respectively; *q*_e,1_ and *q*_e,2_ (mg/g) are the amount of adsorption simulated by the pseudo-first-order and pseudo-second-order, respectively; *F*_fast_ and *F*_slow_ are the mass fractions of “fast” and “slow” compartments, and *F*_fast_ + *F*_slow_ = 1; *k*_fast_ and *k*_slow_ (1/h) are the adsorption rate constants for “fast” and “slow” compartments, respectively; *k*_p_ (mg/g min^0.5^) represents the intraparticle diffusion rate constant; *C* is the adsorption constant. 

The Boyd model is expressed as Equations (6) and (7):

(6) Bt=−0.4977−ln(1−F)

(7) $$F=1-\frac{6}{\pi^{2}}\overset{\infty }{\underset{m=1}{\sum }}\frac{1}{m^{2}}\exp\left[\frac{-D_{i}\pi^{2}m^{2}t}{r^{2}}\right]=\frac{q_{t}}{q_{e}}$$

**Figure 6:**
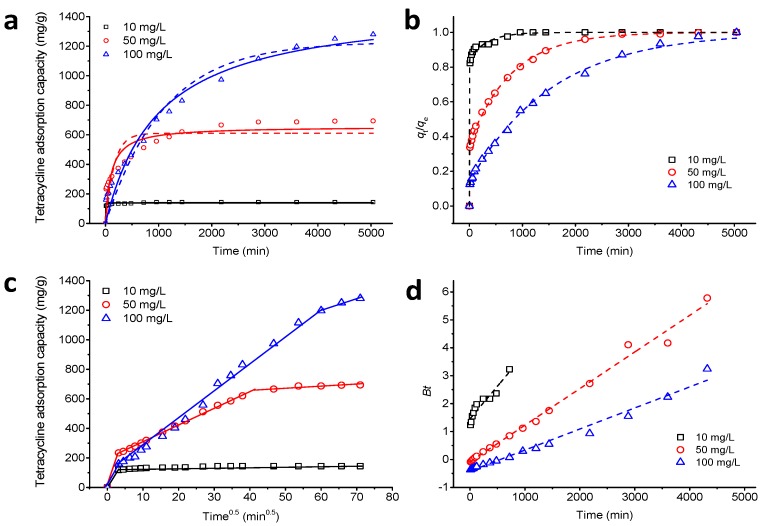
Kinetics of TC adsorption onto Fe_3_O_4_@GO at various TC concentration by fitting (**a**) pseudo-second-order model (dashed lines) and pseudo-second-order model (solid lines), (**b**) two-compartment model, (**c**) intraparticle diffusion model, and (**d**) Boyd model, respectively: pH = 4.0, *m*/*V* = 70 g/L, *C*_0(TC)_ = 10, 50, and 100 mg/L, *T* = 303 K.

*F* represents the adsorption conversion rate at time *t*, and *Bt* is the mathematical function of *F*. The adsorption amount of TC on Fe_3_O_4_@GO as a function of time is shown in Figure 6a. When the initial concentrations are 10 and 50 mg/L, the adsorption rates of TC are fast in the first 12 and 48 h, respectively. Afterward, the reaction rate remains unchanged with further reaction time. When the initial concentration of TC is 100 mg/L, the adsorption rate of TC is slow, and the adsorption equilibrium is reached at 84 h. Figure 6a shows the fittings for nonlinear pseudo-first-order kinetics (dashed line) and pseudo-second-order kinetics (solid line) for the TC adsorption experimental data. The kinetics parameters calculated from the model are shown in Table 1. It can be seen from the figure and table that the pseudo-second-order kinetics model is more suitable for describing the adsorption kinetics than the pseudo-first-order kinetics model, which indicates that the adsorption of TC on Fe_3_O_4_@GO involves chemisorption [48]. Fan et al. [49] also reported that the pseudo-second-order kinetics model better explained the adsorption of tetracycline onto hazelnut shell derived activated carbons.

The adsorption process of TC on Fe_3_O_4_@GO includes two compartments in a two-compartment model, namely, “fast” and “slow” compartments. As shown in Figure 6b and Table 1, the two-compartment model fits the kinetics data for TC adsorption at different concentrations, with a fitting coefficient between 0.996 and 0.999. When the initial concentration is 10 or 50 mg/L, the value of *F*_fast_ is greater than that of *F*_slow_, indicating that the fast adsorption process dominates the TC adsorption. When the initial concentration is 100 mg/L, the slow adsorption process dominates. The removal rates of TC in the fast stage are 97.16%, 70.34%, and 39.01%, respectively, for initial concentrations of 10, 50, and 100 mg/L at shorter exposure times, such as 12 h. 

The reason for this is that at a high initial concentration, the main adsorption process in the beginning is physisorption. Afterward, a portion of the TC was removed by Fe_3_O_4_@GO via a chemisorption process, in which the reaction is slow, and the time to reach adsorption equilibrium was extended [43]. The adsorption data of TC on Fe_3_O_4_@GO were also analyzed using an intraparticle diffusion model. The results are shown in Figure 6c. The segmental linear regression analysis of the data shows that the *q*_t_ vs. *t*_0.5_ curves have three different segments. The first segment represents film diffusion. The second segment describes the adsorption process, in which diffusion within the particles is the rate-limiting step [50]. The straight line in the third region represents the adsorption-desorption equilibrium section. Thus, during the adsorption process, film diffusion and intraparticle diffusion exist simultaneously, and intraparticle diffusion is not the only rate-limiting step in the overall adsorption process. To determine the rate diffusion mechanism that controls the whole adsorption process, the experimental data were fitted using the Boyd diffusion model, as shown in Figure 6d. The *B*t vs. *t* plot is a straight line that does not pass through the origin, indicating that the adsorption rate limiting step is film diffusion [51]. The mass transfer of TC from solution to Fe_3_O_4_@GO is affected by factors such as the rate of oscillation, the particle size of the adsorbent, the concentration of adsorbent, and the affinity of the adsorbent to the contaminant. In this study, film diffusion is the adsorption rate-limiting step, indicating the low mixing rate of the system, the small particle size of Fe_3_O_4_@GO, the low dosage of adsorbent and the high affinity between the adsorbent and adsorbate [47].

### 3.6. Adsorption Isotherm

To study the adsorption efficiency, the experimental data were simulated by Langmuir, Freundlich, and Temkin isothermal models [52]. The equation for the Langmuir model is represented as follows:

(8) $$q_{e}=\frac{q_{\max}K_{L}C_{e}}{1+K_{L}C_{e}}$$

The Freundlich isothermal model is given by Equation (9):

(9) $$q_{e}=K_{F}C_{e}^{1/n}$$

Temkin isothermal model can be represented by Equation (10):

(10) $$q_{e}=\frac{RT}{b_{T}}\ln\left(a_{T}C_{e}\right)$$

where, *K*_L_ (L/mg) is the Langmuir constant; *q*_e_ (mg/g) and *C*_e_ (mg/L) are the amount of adsorption and concentration of TC at equilibrium, respectively; *q*_max_ (mg/g) is the maximum adsorption capacity; *K*_F_ and *n* are the constants of Freundlich model; *R* (8.314 × 10^−3^ kJ/mol K) is the gas constant; *T* (K) is the temperature; *a*_T_ (L/g) and *b*_T_ (kJ/mol) are the Temkin model constants.

**Figure 7:**
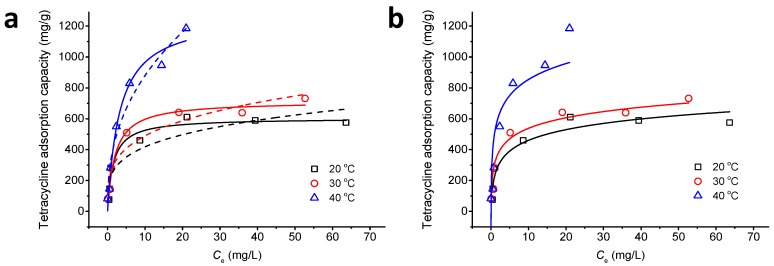
(**a**) Langmuir (solid lines) and Freundlich (dashed lines) and (**b**) Temkin adsorption isotherms for TC adsorption onto Fe_3_O_4_@GO at various temperatures. pH = 4.0, *m*/*V* = 70 g/L, *C*_0(TC)_ = 10 mg/L, *T* = 303 K, *t* = 24 h.

Figure 7 shows the adsorption isotherms of Fe_3_O_4_@GO and TC at different temperatures (20, 30, and 40 °C) and with different initial concentrations of TC (5, 10, 20, 40, 60, 80, and 100 mg/L). The relevant parameters are shown in Table 2. Increasing the initial concentration of TC raises the Fe_3_O_4_@GO adsorption capacity, which may be due to the greater driving force for diffusion from the solution to the adsorption sites at higher concentrations of TC. It can be seen from Figure 7 and Table 2 that the Langmuir model is more consistent with the experimental data than the Freundlich and Temkin models in the temperature range studied. The maximum equilibrium adsorption capacity of TC increases with increasing temperature (the adsorption rates are 603.74, 713.88, and 1272.45 mg/g at 20, 30, and 40 °C, respectively) because of the improved binding between TC and the active sites on Fe_3_O_4_@GO at higher temperatures, which also indicates that the adsorption process is an endothermic reaction. The *q*_max_ is 603.74 mg/g at 20 °C, which is higher than that of graphene oxides (313 mg/g at 30 °C and pH = 3.6) [15], reduced graphene oxides (219.10 mg/g at 25 °C and pH = 6) [4], and hazelnut shell derived activated carbon (312.5 mg/g at 20 °C and pH = 5) [49], indicating that the Fe_3_O_4_@GO is a promising material for the efficient removal of TC.

### 3.7. Desorption Experiment

**Figure 8:**
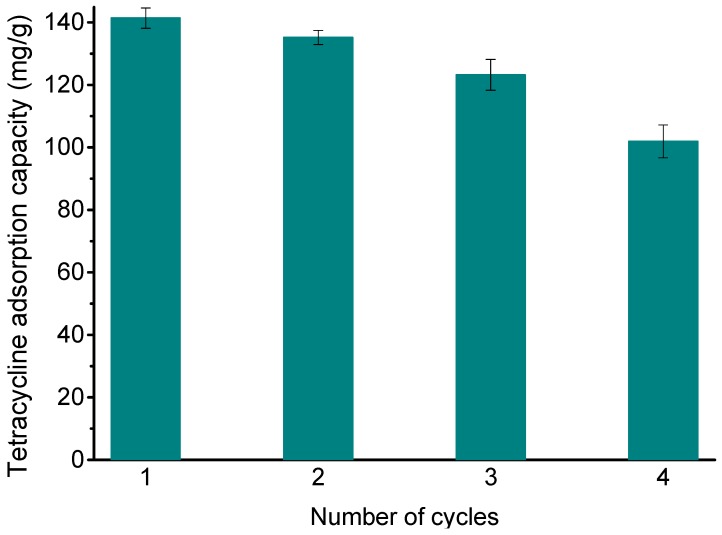
Reusability of the Fe_3_O_4_@GO for TC removal.

Regeneration is key to the use of adsorbents in practical applications. As shown in Figure 8, the adsorption amount of TC on Fe_3_O_4_@GO decreased by 27.90% after four cycles of adsorption-desorption compared with the first adsorption, thus maintaining a high adsorption capacity. It is shown that Fe_3_O_4_@GO has excellent stability and regeneration properties, and it has potential as an adsorbent for removing antibiotics in water.

## 4. Conclusions

According to the results of HRTEM, FTIR, XRD, magnetic hysteresis measurement, TG-DSC, and XPS, Fe_3_O_4_@GO composites were successfully prepared. Fe_3_O_4_@GO shows superparamagnetism and can be separated by a magnet after the adsorption process. The pH value affects the protonation of the surface groups of the adsorbent, resulting in a change in surface charges available and affecting the chemical form of TC in the solution; these factors finally result in a decrease in the adsorption of TC by Fe_3_O_4_@GO with increasing pH. The TC can form inner-sphere complexes with the surfaces of Fe_3_O_4_@GO. HA can react with Fe_3_O_4_@GO and TC, which in turn affects the adsorption process. The adsorption kinetics studies show that the kinetics data conform to the pseudo-second-order kinetics model. The results obtained using the two-compartment model show that when the initial adsorption concentration is 10 or 50 mg/L, the fast adsorption process dominates TC adsorption. When the initial concentration is 100 mg/L, the slow adsorption process dominates. During adsorption, film diffusion and intraparticle diffusion occur simultaneously, and film diffusion is the adsorption rate-limiting step. The isotherm experimental data are consistent with the Langmuir model, and the adsorption reaction is an endothermic process. Fe_3_O_4_@GO has a high adsorption capacity even after 4 cycles of adsorption, indicating that it has an excellent regeneration performance. Based on these results, Fe_3_O_4_@GO can be used as an adsorbent to remove TC from water.

## Figures and Tables

**Table 1:** Kinetic parameters for adsorption of TC onto Fe_3_O_4_@GO.

Models	Parameters	Temperature		
		20 °C	30 °C	40 °C
Pseudo-first-order	*k*_1_ × 10^2^ (1/min)	1.76 × 10^−1^	6.29 × 10^−3^	9.14 × 10^−4^
	*q*_e,1_ (mg/g)	138.27	610.88	1229.09
	*R*^2^	0.951	0.746	0.941
Pseudo-second-order	*k*_2_ (g/mg min)	2.69 × 10^−3^	1.45 × 10^−5^	6.71 × 10^−7^
	*q*_e,2_ (mg/g)	140.76	657.08	1492.21
	*R*^2^	0.977	0.857	0.956
Two-compartment	*F*_fast_	0.15	0.36	0.86
	*F*_slow_	0.85	0.64	0.14
	*k*_fast_ (1/min)	332.21	4.11	1554.70
	*k*_slow_ (1/min)	7.73 × 10^−2^	793.97	5.22
	*R*^2^	0.996	0.999	0.997

**Table 2:** The isotherm parameters for TC adsorption onto Fe_3_O_4_@GO.

Models	Parameters	Temperature		
		20 °C	30 °C	40 °C
Langmuir Isotherm	*q*_max_ (mg/g)	603.74	713.88	1272.45
	*K*_L_ (L/mg)	0.67	0.51	0.31
	*R*^2^	0.950	0.970	0.974
Freundlich Isotherm	*n*	4.03	3.80	2.49
	*K*_F_	236.16	267.11	348.74
	*R*^2^	0.835	0.921	0.957
Temkin Isotherm	*a*_T_ (L/g)	9.96	23.51	30.69
	*b*_T_ × 10^2^ (KJ/mol)	2.44	2.56	1.75
	*R*^2^	0.919	0.917	0.757
